# New Perspectives on the Role of Integrin-Linked Kinase (ILK) Signaling in Cancer Metastasis

**DOI:** 10.3390/cancers14133209

**Published:** 2022-06-30

**Authors:** Paul C. McDonald, Shoukat Dedhar

**Affiliations:** 1 Department of Integrative Oncology, BC Cancer Research Institute, Vancouver, BC V5Z 1L3, Canada; pmcdonal@bccrc.ca; 2 Department of Biochemistry and Molecular Biology, University of British Columbia, Vancouver, BC V6T 1Z3, Canada

**Keywords:** integrin-linked kinase, cell signaling, invasion, colonization, metastasis, cell plasticity, epithelial mesenchymal transition, neutrophil extracellular trap, therapeutic resistance

## Abstract

**Simple Summary:**

Today, the vast majority of deaths from cancer are due to cancer metastasis. Metastasis requires that cancer cells escape from the initial tumor, travel through blood vessels, and form new tumors in distant host tissues. Integrin-linked kinase (ILK) is overexpressed by many types of cancer cells and provides both structural and signaling functions that are important for successful metastasis. Here, we discuss recent findings that show how ILK is involved in promoting physical changes important for cell motility and invasion, and how ILK relays signals to other machinery components during metastasis, including interactions with components of the immune system and communication between cancer cells and normal cells, to affect the process of metastasis. We also discuss the contribution of ILK to therapeutic resistance and examine efforts to target ILK for the treatment of metastatic disease.

**Abstract:**

Cancer metastasis is a major barrier to the long-term survival of cancer patients. In cancer cells, integrin engagement downstream of cell-extracellular matrix (ECM) interactions results in the recruitment of cytoskeletal and signaling molecules to form multi-protein complexes to promote processes critical for metastasis. One of the major functional components of these complexes is Integrin Linked Kinase (ILK). Here, we discuss recent advances in our understanding of the importance of ILK as a signaling effector in processes linked to tumor progression and metastasis. New mechanistic insights as to the role of ILK in cellular plasticity, epithelial mesenchymal transition (EMT), migration, and invasion, including the impact of ILK on the formation of invadopodia, filopodia-like protrusions (FLPs), and Neutrophil Extracellular Trap (NET)-induced motility are highlighted. Recent findings detailing the contribution of ILK to therapeutic resistance and the importance of ILK as a potentially therapeutically tractable vulnerability in both solid tumors and hematologic malignancies are discussed. Indeed, pharmacologic inhibition of ILK activity using specific small molecule inhibitors is effective in curtailing the contribution of ILK to these processes, potentially offering a novel therapeutic avenue for inhibiting critical steps in the metastatic cascade leading to reduced drug resistance and increased therapeutic efficacy.

## 1. Introduction

Cancer metastasis continues to pose the most significant barrier to the long-term survival of cancer patients, with over 90% of cancer deaths attributable to the impacts of metastatic disease [1]. Globally, metastasis proceeds via three broad phases of dissemination, dormancy, and colonization/outgrowth [2]. Dissemination involves the initial movement of tumor cells from the primary tumor to both regional and distal sites. Importantly, this phase may continue as long as the primary tumor is present and capable of releasing circulating tumor cells (CTCs). Dissemination requires extravasation from capillaries, infiltration of the host parenchyma, and establishment of cells within the tissue niche [2]. At this point, properties of the tissue microenvironment at the site of metastasis, including physical, metabolic, and immune barriers imparted by the host stroma, may propel disseminated cancer cells into dormancy, a state of proliferative quiescence where cells are in equilibrium with environmental factors, including host immunity. Ultimately, the inability of the immune system and environmental niche to keep dormant cancer cells in check leads to colonization and the outgrowth of macrometastases [2]. Importantly, all three phases of metastasis may exist simultaneously within an individual patient and clinically detectable metastases may occur months, years, or decades after initial diagnosis and therapy.

Cell-extracellular matrix (ECM) interactions play critical roles in all three phases of metastasis and involve the interaction of integrin receptors with distinct components of the ECM. Integrin engagement, in turn, results in the recruitment of actin cytoskeletal and signaling molecules to form multi-protein complexes at the site of these interactions to promote cell growth, survival, migration, and invasion. One of the major functional components of these complexes is Integrin Linked Kinase (ILK) and its immediate interactors particularly interesting new cysteine histidine-rich-protein (PINCH) and Parvins: the IPP complex [3,4].

The past decade has seen dramatic advances in our understanding of the importance of ILK as a signaling effector in several processes linked to tumor progression and metastasis. In addition to its importance in the process of anoikis in tumor progression [5], several studies have provided new mechanistic insights as to the role of ILK in several steps of the metastatic cascade, including extravasation, colonization, and dormancy. New discoveries pertain to ILK-mediated signaling in cellular plasticity, epithelial mesenchymal transition (EMT), migration, and invasion, including the impact of ILK on the formation of invadopodia and filopodia-like protrusions (FLPs). Increasing evidence also points to the importance of ILK in leukemia, particularly in interactions between leukemic stem cells and the bone marrow matrix environment, suggesting a role of ILK in altered niche environments. Furthermore, there is mounting evidence that ILK, through its role in the regulation of signaling pathways, contributes to therapeutic resistance, suggesting that ILK is a potentially therapeutically tractable vulnerability. Indeed, several studies have shown that pharmacologic inhibition of ILK using specific small molecule inhibitors is effective in curtailing the contribution of ILK to these processes, potentially offering a novel therapeutic avenue for curtailing critical steps in the metastatic cascade leading to reduced drug resistance and increased therapeutic efficacy.

## 2. ILK Functions as a Signaling Node within Focal Adhesions

**Figure 1:**
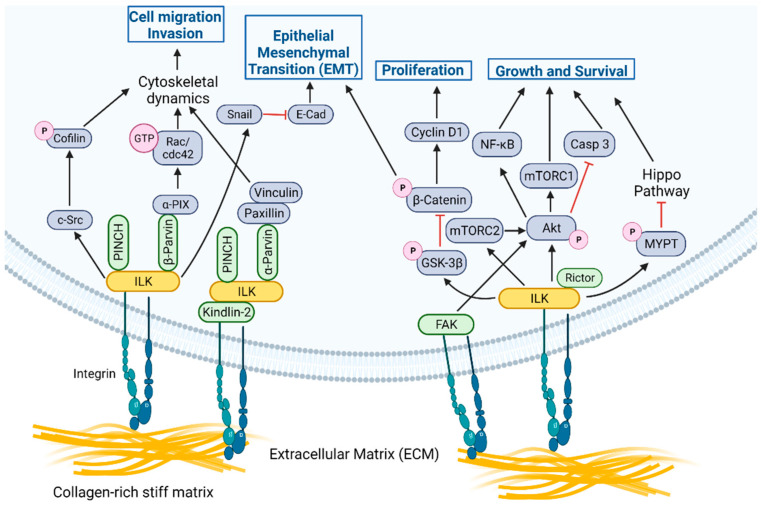
ILK functions as a signaling node at focal adhesions in cells. The ILK-PINCH-Parvin (IPP) complex acts as a structural and signaling hub downstream of integrin-mediated engagement with collagen-rich, stiff ECM [12] to promote processes critical for metastasis, including cytoskeletal dynamics, EMT, migration, and invasion. ILK also promotes phosphorylation of key target proteins essential to processes critical for cancer progression, including survival, proliferation, migration, invasion, and EMT. For the purposes of illustration, we have shown ILK or FAK associated with integrins, but these signaling axes are not mutually exclusive and could be associated in the same complex. Created with Biorender.com. Available online: https://biorender.com/ (accessed on 21 June 2022).

ILK is a multi-functional signal transduction effector, which, coupled with PINCH1/2 and α/β/ϒ-Parvin proteins, forms the tripartite ILK-PINCH-Parvin (IPP) complex that acts as a structural and signaling hub [6,7]. IPP is tightly regulated and the suppression of expression of any individual component results in proteasome-mediated degradation of the other components. IPP is recruited to focal adhesions (FA) where ILK associates with the cytoplasmic domain of β-integrins (Figure 1), either directly [8,9] or through proteins such as Kindlin-2, which binds directly to integrins and to ILK [10]. Interestingly, while Talin is also a well-recognized component of FA [11], clear evidence that Talin directly associates with ILK in this context is lacking. Integrin-ILK engagement forms physical interactions within cells that coordinate and facilitate reciprocal signaling networks between the intracellular and extracellular environments (Figure 1). These networks regulate a diverse array of cellular processes, including cell growth, proliferation, differentiation, survival, migration, and invasion. Downstream of the IPP in FA, ILK participates in several key signaling pathways central to these processes [8]. For example, ILK promotes the activation of c-Src, resulting in Src-mediated phosphorylation of cofilin and subsequent actin polymerization. ILK activates α-PIX (PAK-interactive exchange factor), which, in turn, activates Rac/cdc42 to promote motility. ILK also interacts with Nck-2 through PINCH within the IPP, connecting integrin-mediated signaling with growth factor-mediated signaling inputs. Finally, while both focal adhesion kinase (FAK) and ILK are localized to FA and are key transducers of integrin signaling, there is currently no evidence of a direct interaction and no studies demonstrating that either kinase can phosphorylate the other, although they may cooperate to transduce signals downstream of integrins in certain contexts (Figure 1) (discussed in Section 5 on therapeutic resistance). Thus, as a component of the IPP, ILK participates in several protein-protein interactions that affect downstream signaling pathways.

In addition to its role as part of the IPP, ILK promotes phosphorylation of key target proteins essential to processes critical for cancer progression, including survival, proliferation, migration, invasion, and EMT (Figure 1). Downstream targets of ILK include Akt, rapamycin-insensitive companion of mammalian target of rapamycin (Rictor), glycogen synthase kinase-3 beta (GSK3β), β-catenin, myosin phosphatase target subunit 1 (MYPT-1) in the Hippo pathway, and myosin light chain (MLC) [7,8]. The ability of ILK to regulate phosphorylation of components of the phosphoinositide 3-kinase/mammalian target of rapamycin (PI3K/mTOR) signaling complex, such as Akt and Rictor, is critical in promoting tumor cell survival [13,14,15]. ILK promotes phosphorylation of and subsequent inactivation of GSK3β, resulting in regulation of cyclin D1 and activation of transcription factors (TF), including activator protein-1 (AP-1), β-catenin/T-cell factor (Tcf), and cAMP response element-binding protein (CREB). β-catenin stabilization results in the regulation of proliferation and migration. Intriguing recent findings in the context of familial exudative vitreoretinopathy (FEVR), a disease involving defective Wnt-β-catenin signaling, further illustrate the strong functional link between ILK and Wnt signaling [16]. Inactivation of ILK in postnatal endothelial cells (ECs) reduces β-catenin levels in vitro and in vivo, leads to modulation of lymphoid enhancer binding factor 1 (LEF1) expression in the retinal vasculature and results in the perturbation of retinal angiogenesis, similar to phenotypes observed in models and patients with FEVR [16]. The defects are further phenocopied by the inactivation of the ILK interactor, α-Parvin, implicating the IPP complex and linking ILK-mediated cell-matrix interactions and Wnt signaling [16]. It has also been observed that LIM domain-containing protein 2 (LIMD2), a LIM-domain only protein specifically expressed in metastatic lesions [17], directly binds the kinase domain of ILK and increases ILK activity, leading to increased migration and invasion by cancer cells [18]. The ILK/LIMD2 complex and the regulation of ILK activity by LIMD2 suggest a potential mechanism for upregulation of ILK activity specifically in metastatic cancer cells [18]. ILK signaling also regulates the Hippo tumor suppressor pathway, since ILK-mediated phosphorylation of MYPT-1 results in negative regulation of the Hippo pathway, which has effects on cancer cell growth, invasion, and drug resistance pathways [19].

## 3. Role of ILK Signaling in Invasive Structures, Invasion, Colonization, and Dormancy

### 3.1. Invasive Structures

ILK is an established linker of integrins to the actin cytoskeleton. When cells are attached and spread on ECM, ILK localizes to focal adhesions [8,11]. However, when cells are induced to migrate and invade, ILK localizes to motility and invasion-specific structures such as lamellipodia [20,21], FLPs [22,23], and invadopodia [24,25].

Invasion by cancer cells requires the presence and activity of invadopodia, which are specialized, actin-rich membrane protrusions that function to degrade surrounding ECM, facilitating cell motility. The formation and functional organization of invadopodia is complex and requires the presence of a filamentous actin (F-actin)-cortactin core surrounded by actin regulatory proteins, including actin related protein 2/3 complex (ARP2/3), neuronal Wiskott-Aldrich Syndrome protein (N-WASP), and cofilin, the presence of proteins involved in adhesion, scaffolding, and signaling, and a continuous supply of proteases. In this context, integrin-ILK signaling is critical to the effective formation and function of invadopodia.

**Figure 2:**
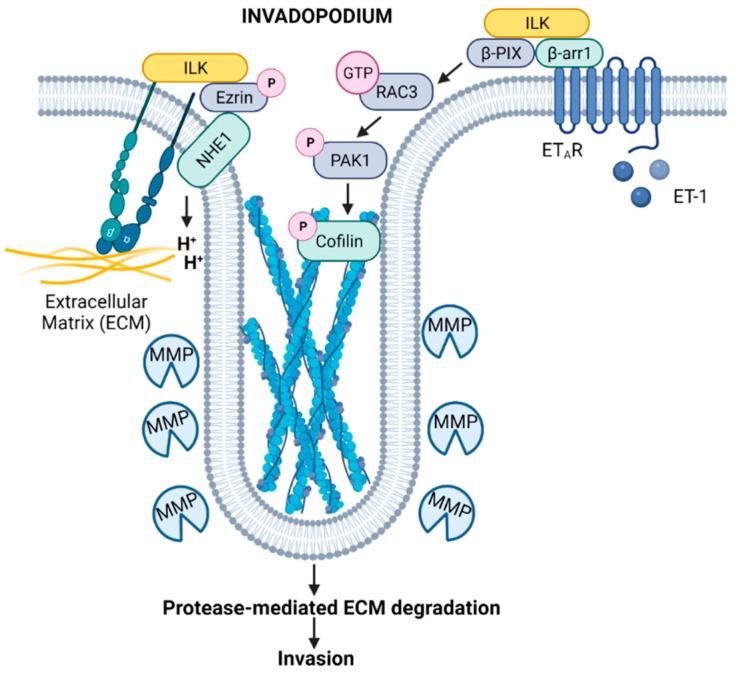
Model depicting the contributions of ILK signaling to the function of invadopodia. Integrin-induced activation of ILK drives NHE1-mediated scaffolding and acidification functions through an ILK/p-ezrin/NHE1 complex [25], while ET-1-mediated activation of a β-arr1/ILK/β-PIX platform leads to Rac activation and phosphorylation of cofilin [24], promoting invadopodium-dependent ECM proteolysis and degradation. These ILK signaling platforms may potentially cooperate to expedite ECM degradation and invasion by cancer cells. Created with Biorender.com. Available online: https://biorender.com/ (accessed on 2 June 2022).

Genetic depletion of ILK has revealed that ILK is required for the formation of adhesion rings that promote structural maturation, recruitment of the ILK-binding vesicular adaptor protein IQ motif containing GTPase activating protein (IQGAP), and accumulation of membrane type 1-matrix metalloproteinase (MT1-MMP; also known as MMP-14) at invadopodia, all of which are important for the maturation and degradative functions of these structures [26]. ILK has also been linked to Na^+^/H^+^ exchanger type 1 (NHE1)-induced invadopodia activation through a β1-integrin stimulated phosphorylated (p)-ezrin/ Na+/H+ exchange regulatory factor 1 (NHERF1)/NHE1 signaling complex (Figure 2). Investigation of protein complexes using approaches such as proximity ligation assays, in the context of inhibition of ILK, demonstrates that, in breast and prostate cancer cells, ILK interacts with β1-integrin, NHE1, and the scaffold proteins p-ezrin and NHERF1 to form a β1-integrin/ILK/p-ezrin/NHE1/p-NHERF1 complex to regulate NHE1-mediated ECM proteolysis and cell invasion, promoting an invasive phenotype [25].

Furthermore, recent studies in serous ovarian cancer (SOC) indicate that ILK may function as a link between proteolytic and adhesive signaling, connecting ILK cytoskeletal dynamics and cell invasion processes mediated by invadopodia [24]. Engagement of endothelin A receptor (ET_A_R) triggers activation of β-arrestin1 (β-arr1), which interacts with an ILK/β-PIX complex. The β-arr1/ILK/β-PIX platform regulates the activity of Rac3, which then phosphorylates p21-activated kinase 1 (PAK1) and cofilin, promoting invadopodium-dependent extracellular matrix proteolysis and invasion (Figure 2) [24]. Moreover, ET_A_R-mediated ILK/β-PIX/Rac3 signaling regulates the interaction of SOC cells and mesothelial cells, promoting SOC adhesion and transmigration, and concomitant high expression of ET_A_R and ILK in SOC patients is correlated with shorter survival [24].

### 3.2. Mechanotransduction, Matrix Stiffness, and Invasion

Several recent studies have uncovered a dynamic interplay between tissue stiffness and ILK-mediating signaling. The relative compliance of the extracellular matrix in the microenvironment of both the primary tumor and of metastases has a dramatic influence on the phenotypic properties of cancer cells. The substrata of primary solid tumors are generally stiffer than adjacent normal tissue, while breast cancer metastases often develop in tissues of less stiff composition, such as the lung, liver, and brain. A series of studies employing engineered synthetic culture substrata that emulate the mechanical properties of relatively soft, compliant tissue matrices and stiff matrices common in solid tumors have revealed a critical role of ILK as a mechanotransducer in promoting metastatic progression (Figure 1) [12,27,28,29,30,31].

In primary tumors, stiff tumor microenvironment (TME) matrices promote EMT, while softer substrata induce apoptosis. Mammary epithelial cells (MEC) cultured on stiff matrices and/or in the presence of ILK expression exhibit robust focal adhesions coupled with reduced adherens junctions and undergo EMT in response to transforming growth factor beta (TGF-β), while MECs depleted of ILK expression show decreased FA activation, including levels of activated FAK and paxillin, increased cell-cell adhesions and undergo TGF-β mediated apoptosis, similar to MECs cultured on soft matrix [12]. These data suggest that environmental stiffness signals through ILK to dial in cell-cell and cell-matrix interactions to control the phenotypic response to TGF-β [12].

In contrast to normal mammary tissue, the TME of breast tumors is hypoxic and mechanically stiff. The relative stiffness of the ECM and the presence of hypoxia, two critical features of the TME in solid tumors such as breast cancer, interact through ILK signaling to synergistically potentiate cancer stem cell (CSC) marker expression, tumor growth, and metastasis [31]. Stiff matrices, coupled with hypoxia result in a synergistic increase in ILK expression, coupled with increased expression of CSC markers and increased motility by breast cancer cells, suggesting that these environmental stressors potentiate ILK signaling to promote metastasis [31]. Depletion of ILK expression in the context of a stiff matrix reduces the expression of β1-integrin and CSC markers, and impacts secondary mammosphere formation, suggesting the importance of ILK for the induction of CSC marker expression and behavior [31]. In contrast, constitutive overexpression of ILK promotes the development of CSCs in soft, normoxic conditions [31]. Tumor formation and metastasis increase with tissue stiffness in ovo and ILK depletion in this context abolishes the invasiveness and metastatic potential of breast cancer cells [31]. Increasing matrix stiffness promotes β1-integrin-induced signaling through the ILK/PI3K/Akt pathway to control CSC development and, simultaneously induces a feed-forward loop in which the activation of ILK-mediated signaling induces increased expression of β1-integrin and ILK [31].

Interestingly, while soft matrices result in apoptosis of MECs downstream of TGF-β stimulation [12], breast cancer cells cultured in soft microenvironments, such as those found in tissues such as lungs, bone, brain, and liver, upregulate autophagy processes to remain quiescent. Ectopic expression of ILK in epithelial breast cancer cells decreases the number and volume of autophagosomes when cells are grown on soft substrata, relative to stiff matrices, suggesting a role of ILK in regulating autophagy during dormancy [29].

### 3.3. Extravasation and Colonization

**Figure 3:**
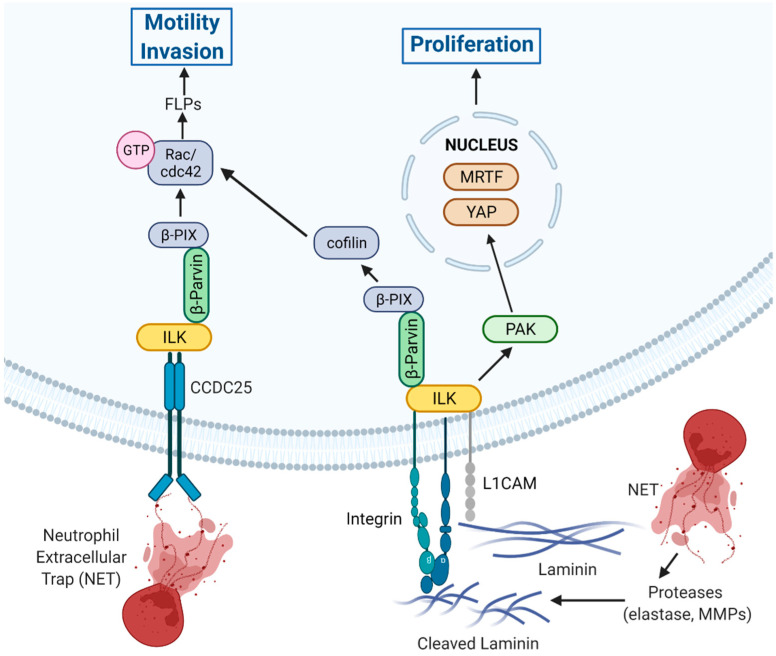
New insights into the role of ILK signaling in promoting extravasation and colonization. ECM-mediated engagement of integrins and NET-mediated engagement of CCDC25 potentially cooperate to engage ILK/β-parvin signaling axis functions to promote filopodia-like protrusions (FLPs) and promote motility and invasion [22,33]. L1CAM also cooperates with β1-integrins to engage ILK, leading to regulation of FLPs as well as to PAK-mediated activation of YAP and MRTF to initiation of proliferation and metastatic outgrowth [32]. Created with Biorender.com. Available online: https://biorender.com/ (accessed on 2 June 2022).

A particularly interesting aspect of ILK signaling is its function as a major component of a central pathway that enables colonization by disseminated cancer cells (DCCs) immediately subsequent to extravasation and during exit from latency or dormancy. The eventual growth of macro-metastases requires initial growth within the perivascular niche and involves L1 cell adhesion molecule (L1CAM)-dependent vascular co-option, an essential source of mechanotransduction inputs required for metastatic outgrowth [32]. Subsequent to engagement, L1CAM cooperates with β1-integrins and engages ILK to initiate downstream signaling to p21-activated kinase (PAK) to activate the transcription factors yes-associated protein (YAP) and myocardin-related transcription factor (MRTF) and initiate metastatic outgrowth (Figure 3) [32].

The studies have also determined that the L1CAM-ILK-YAP signaling axis is critical for colonization at distant sites in breast cancer cells [32]. Knockdown of ILK expression in breast cancer cells suppresses pericytic spreading of disseminated cancer cells (DCC) and inhibits metastatic colonization of the brain, lungs, and bone [32]. Depletion of ILK following administration of cells in models of experimental metastasis inhibits metastatic colonization, demonstrating a requirement for ILK for cell spreading and outgrowth of metastases [32]. Knockdown of L1CAM reduces ILK-dependent F-actin-rich filopodia-like protrusions (FLPs) and ILK knockdown suppresses nuclear localization of YAP [32].

The extravasation of DCCs into the parenchyma of host tissues, colonization, and proliferation of disseminated cells is required for the growth of macrometastases. Colonization requires that disseminated cells establish β1-integrin-mediated adhesions with the ECM, a process facilitated by the formation and action of actin-rich FLPs, which enable cancer cells to interact with surrounding ECM to regulate initial proliferation key to colonization and outgrowth [22,23]. Initial proliferation subsequent to dissemination is governed, in part, by the collaborative actions of FLP formation and retention that increase FLP abundance. Studies in colonization-competent cancer cells have demonstrated the importance of an ILK/β-parvin/cofilin signaling axis in regulating FLP stability [22]. The ILK/β-parvin interaction signals downstream to β-PIX/cdc42/PAK to control the LIM domain kinase 1 (LIMK)/cofilin pathway to suppress cofilin-dependent cleavage of actin fibers in FLPs, leading to increased lifetime and abundance of FLPs (Figure 3) [22]. Inhibition of this signaling axis reduces cancer cell proliferation in vitro and suppresses lung metastasis in vivo, demonstrating the importance of this ILK-mediated signaling cascade in controlling metastatic propensity. Moreover, the increased frequency of tumor-initiating cells is due, in part, to increased FLP stability, which is due to elevated ILK/cofilin signaling [22]. As well, EMT induced by Twist or Snail increases expression of the pathway components, including ILK, demonstrating a link between EMT, tumor-initiating ability, and ILK signaling [22,23].

It is interesting to note that, in the context of invasion, the role of ILK in FA, invadopodia, and FLPs has been documented largely in cell culture models. In contrast, there is a paucity of information surrounding the specific localization of these invasive processes in tissues and tumors in vivo, and this remains an important area of future investigation.

### 3.4. ILK as a Key Component of Neutrophil Extracellular Trap (NET) Mediated Invasion and Metastasis

NETs are structures of decondensed genomic DNA decorated with histones and proteases such as myeloperoxidase (MPO) and neutrophil elastase (NE), which are produced and released by neutrophils at sites of bacterial infiltration and inflammation through a process known as NETosis [34]. Interestingly, recent evidence suggests that NETosis is a critical contributor to cancer progression. In cancer patients, the presence of increased NETs in the tumor and in the circulation is correlated with both poor prognosis and aggressive tumor behavior [35,36]. NETs have been shown to play a role in primary tumor growth mediated by hypoxia [37] and in response to tumor cell-derived chemokines and cytokines [38]. Furthermore, tumors can directly stimulate NETosis [35,38,39,40].

Beyond contributing to the growth of primary tumors, studies suggest that NETs are an integral component of metastatic progression. NETs play a role in arresting circulating tumor cells (CTCs) in distant organs and facilitate the outgrowth of metastases [35,41]. Recent work also suggests that NET-associated proteases function to facilitate the remodeling of the ECM within the metastatic niche, leading to the reactivation of dormant cancer cells [42]. Furthermore, analysis of primary tumors, plasma, and metastases from breast cancer patients for the NET markers MPO and citrullinated histone H3 (cit-H3) revealed an association between increased levels of NET-DNA in the liver and plasma coupled with increased metastasis, suggesting a role of NETs in metastatic progression and highlighting the potential of circulating NET-DNA as a predictive biomarker of hepatic metastasis [33].

Recently, data has emerged in breast cancer demonstrating a mechanism by which NETs mediate disseminated cancer cells to become metastatic (Figure 3). Interrogation of breast cancer cells for a putative NET-DNA receptor capable of mediating signaling resulted in the identification of a coiled coil domain containing protein 25 (CCDC25) [33]. Subsequent depletion of CCDC25 inhibited NET-DNA stimulated cytoskeletal remodeling, migration, and proliferation of MDA-MB-231 cells in vitro, and reduced liver metastases in vivo [33]. Further investigation of interacting proteins downstream of CCDC25 that could facilitate signal transduction identified ILK as being recruited by NET-DNA to CCDC25 [33]. NET-DNA-mediated stimulation of breast cancer cells resulted in activation of a NET-CCDC25-ILK signaling axis resulting in the activation of the migration/invasion promoting Rho family GTPases, Rac, and cdc42, while depletion of CCDC25, ILK, or β-parvin reduced Rac1/cdc42 activation (Figure 3) [33]. Such findings suggest that upon the interaction of NET-DNA with CCDC25, a portion of the ILK pool may translocate from integrin-containing complexes to CCDC25 complexes, resulting in its altered subcellular localization, activation, and signaling.

In addition to the NET-DNA-CCDC25 complex, studies using patient samples and xenograft models in the setting of breast cancer metastasis and bevacizumab-resistant invasive glioblastoma demonstrated ILK-dependent phosphorylation of c-Met, promoting ligand-independent receptor activation downstream of c-Met/β1-integrin complex formation [43] and high-affinity binding to fibronectin, allowing tumor cells to adapt to TME stressors such as hypoxia and bevaziumab exposure and drive chemotactic and haptotactic invasion [43]. Genetic depletion of ILK expression or pharmacologic inhibition of ILK with an ILK inhibitor, Compound 22, blocked ligand-independent c-Met phosphorylation in the presence of fibronectin [43], and treatment with Compound 22 also reduced adhesion and spreading of U87 glioblastoma cells on fibronectin.

### 3.5. ILK and Extracellular Vesicles

Recent work has uncovered a unique role of ILK signaling in cross-talk between cancer cells and adjacent normal cells mediated by small extracellular vesicles (sEV). sEV, which are produced by cells and package bioreactive molecules for extracellular transport, are important for intercellular communication and are linked to modulation of integrin-dependent adhesion, migration, and regulation of mitochondrial dynamics. Studies have shown that hypoxia stimulates the release of sEV from breast cancer cells and these sEV interact with neighboring normal MEC to drive several malignant changes, including stimulation of ILK-Akt signaling, turnover of FA, and enhanced motility of epithelial cells [44]. Interestingly, ILK-mediated events were identified both in the stimulated normal epithelial cells and in the cancer cell-derived sEV [44,45]. sEV produced by breast cancer cells in hypoxia contain increased amounts of ILK and subsequent interaction of these sEV with normal mammary cells activates NFκB signaling, resulting in inflammatory cytokine release and activation of mitochondrial dynamics leading to increased cell motility, and, in 3-dimensional (3D) mammary acini, to dysregulated proliferation, reduced apoptosis and promotion of EMT [45]. On the flip-side, global RNAseq profiling of sEV-treated normal MEC identified ILK signaling as a major activated pathway, and blocking ILK using genetic and pharmacologic approaches suppressed sEV-induced cell motility [44]. Examination of 3D models of mammary gland formation revealed that treatment with sEV derived from hypoxic cancer cells resulted in ILK upregulation, increased proliferation, decreased apoptosis and increased EMT markers, and pharmacologic inhibition of ILK suppressed EMT markers, including vimentin and N-cadherin.

## 4. Role of ILK Signaling in EMT and the Hippo Pathway

### 4.1. Epithelial Mesenchymal Transition

Epithelial-Mesenchymal Transition (EMT) is a vital process during tumor progression and metastatic spread [2,46], and a key mechanistic process enabling metastatic dissemination. EMT is driven by transcription factors (TFs), including Snail, Slug, ZEB, and Twist, that function to repress epithelial genes and upregulate mesenchymal factors [2,46]. Cells that undergo EMT have an increased capacity to migrate and invade, allowing for dissemination and infiltration of distant organs. Following infiltration and colonization of distant sites, cells may undergo the reverse process, mesenchymal epithelial transition (MET) to enable proliferation and growth [2,46]. Importantly, the transition from epithelial to mesenchymal states occurs along a highly dynamic continuum and underscores the phenotypic plasticity of cancer cells [46]. It is now well-recognized that ILK and ILK-mediated signaling is centrally involved in the process of EMT by cancer cells.

ILK activity is required for the promotion of EMT by TGF-β1, a potent EMT inducer, in MEC. TGF-β-mediated EMT induces an ILK-Rictor interaction, together with phosphorylation of Rictor at Thr1135 and nuclear translocation of Snail and Slug, all of which are suppressed by depletion of ILK or inhibition of ILK activity [47]. ILK inhibition also partially reverses EMT in MDA-MB-321 breast cancer cells [47]. Increased levels of ILK expression results in upregulation of EMT markers including Snail, Slug, vimentin, and N-cadherin, and suppression of E-cadherin expression, promoting EMT, invasion, and metastasis in oral [48,49], lung [50], and colon cancer [51]. In contrast, suppression of ILK reduces EMT markers and inhibits invasion and metastasis in many cancer types [52,53,54,55]. In melanoma cells, siRNA-mediated depletion of ILK suppresses the expression of N-cadherin, showing that ILK is involved in cadherin switch, a hallmark of EMT [56].

Several ILK-mediated signaling pathways have been implicated in the regulation of EMT in cancer cells. Targeting the ILK signaling axis results in inhibition of phosphorylation of Akt and GSK3β in breast cancer [57], ovarian cancer [58,59], and hepatocarcinoma [60], among others. Proteomic analysis following Twist-mediated induction of EMT in MEC results in activation of FAK/ILK, mitogen-activated protein kinase/extracellular signal-regulated kinase (MAPK/ERK), PI3K/Akt, and Wnt signaling, while depletion of ILK curtails Twist-mediated EMT and invasion [61], and depletion of Twist in breast cancer cells decreases activation of FAK/ILK and downstream signaling, leading to reduced EMT and invasion [61]. Twist also correlates with ILK, Snail, and zinc finger E-box-binding homeobox 1 (ZEB1) in phyllodes tumors of the breast, driving EMT-mediated malignant progression of these tumors [62]. Overexpression of ILK in colorectal cancer cells results in activation of NF-kB and promotion of EMT, while depletion of ILK expression or inhibition of NF-kB suppresses EMT in these cells, indicating a role of ILK-mediated NF-kB signaling in colon cancer [51,63].

### 4.2. Hippo Pathway

It is becoming increasingly clear that ILK is a vital negative regulator of the Hippo tumor suppressor pathway in cancer cells. Importantly, ILK kinase activity is crucial for this functionality. ILK inhibits MYPT-1 through direct phosphorylation, preventing activation of downstream effectors and resulting in nuclear accumulation of YAP [19]. Conversely, genetic suppression of ILK expression or pharmacologic inhibition of ILK activity results in activation of the Hippo pathway, leading to YAP phosphorylation, cytoplasmic sequestration, and inhibition of transcriptional enhanced associate domain (TEAD) transcriptional activity [19]. Moreover, ILK inhibition leads to suppression of tumor growth and inhibition of YAP activation in vivo [19]. These initial findings have fostered further investigations that have identified links between ILK/YAP signaling, EMT, and metastatic progression.

Clinically, high levels of expression of ILK and YAP are correlated with poor prognosis in breast cancer [64,65] and glioma [66], while heightened expression of an ET_A_R/ILK/YAP/AP-1/ZEB1 gene signature is correlated with significantly shortened survival in a cohort of SOC patients [67]. In patients with breast cancer, high levels of expression of ILK and YAP are associated with increased disease stage and lymph node metastases, linking this signaling axis clinically to metastatic progression [64].

The findings of several studies suggest that modulation of ILK expression is a key driver of ILK-mediated regulation of YAP signaling promoting EMT, invasion, and therapeutic resistance. Suppression of TGF-β mediated EMT, migration, and invasion of breast and lung cancer cells by LFG-500, a synthetic anti-inflammatory flavonoid with potential anti-cancer properties, operates through down-regulation of ILK expression and subsequent downstream activation of the Hippo signaling pathway [64]. Studies in SOC further illustrate the link between increased levels of ILK and ILK-mediated negative regulation of the Hippo pathway in cancer cells to promote EMT. In SOC cells, endothelin-1 mediated activation of ET_A_R results in increased expression of ILK and nuclear accumulation of YAP, together with ZEB1 [67], initiating transcriptional activation of ZEB1 and endothelin 1 (ET-1) production and facilitating a feed-forward loop resulting in chronic ET-1-mediated signaling [67] that drives ET-1R/ILK induced EMT, cellular plasticity and invasion [67]. Inhibition of ETR with macitentan suppresses ZEB1/YAP and inhibits SOC progression [67].

ILK/YAP signaling is also implicated in therapeutic resistance. Breast cancer cells cultured on polyacrylamide hydrogels of varying stiffness were observed to show differential levels of ILK expression [65]. Increased expression of ILK on matrices of intermediate stiffness results in inactivation of the Hippo pathway downstream of ILK and nuclear translocation of YAP, while matrices of high or low stiffness result in decreased ILK expression and consequent activation of the Hippo pathway [65]. High levels of ILK expression are associated with YAP-mediated increases in p-glycoprotein (P-gp) expression, leading to chemoresistance to drugs such as doxorubicin [65], while reduced levels of ILK results in increased drug sensitivity [65].

## 5. The Role of ILK Signaling in Therapeutic Resistance

A central feature of metastatic cancer cells is resistance to cancer therapies, a characteristic that is intimately intertwined with cellular plasticity. Recent studies have demonstrated that the expression of ILK modulates the resistance of cancer cells to therapeutic initiatives. Recent unbiased, genome-wide investigations to identify potential targets for combination therapies centered on Src inhibitors identified ILK, in association with α-Parvin and PINCH-1 as a central determinant of sensitivity to the Src/Abl kinase inhibitor, bosutinib in breast cancer [68]. Enhanced sensitivity to bosuntinb in the context of ILK depletion was centered on the inhibition of Src and was associated with cell adhesion defects, leading to increased G1 arrest and apoptosis [68].

ILK signaling has also been implicated in resistance to inhibitors of mTOR. While inhibition of mTOR complex 1/2 (mTORC1/2) results in initial suppression of Akt-mediated survival, sustained inhibitory pressure results in reorganization of integrin-mediated adhesion, stimulation of insulin-like growth factor 1 (IGF-1) receptor/insulin receptor (IGFR/IR)-dependent PI3K activation, and Akt phosphorylation, a process regulated by ILK signaling [13]. Specifically, genetic and pharmacologic perturbation of ILK activity blocks Akt phosphorylation and activation in the presence of mTORC1/2 inhibition, showing that reactivation of Akt is ILK-dependent [13]. Intriguingly, inhibition of mTORC2 also resulted in regulation of Akt phosphorylation by FAK, revealing that, in the context of mTORC2 inhibition, integrin signaling through FA can regulate Akt phosphorylation and activity through both FAK and ILK (Figure 1) [13]. Similarly, depletion of α2 or β1-integrin expression by hepatocellular carcinoma (HCC) cells in the context of IGF-1-driven signaling results in suppression of both ILK and FAK activity, leading to diminished activation of Akt/mTOR signaling and further indicating cooperative signal transduction by ILK and FAK downstream of integrins [69]. Furthermore, inhibition of KRAS or downstream MEK/ERK signaling results in initial reductions in the growth of pancreatic tumor cells, but leads to compensatory activation of Akt through ILK-mediated phosphorylation of Rictor, promoting mTORC2-mediated Akt phorphorylation [15].

In colorectal cancer (CRC), increased ILK expression in patients correlates with markers of EMT and CSCs and is associated with metastasis and chemoresistance. Furthermore, inhibition of ILK in therapy-resistant CRC cells suppresses levels of EMT and CSC markers and sensitizes the cells to 5-fluorouracil (5-FU) and oxaliplatin [70].

ILK signaling has been implicated in the mechanotransduction-mediated regulation of autophagy at sites of distant metastasis in breast cancer. There is also mounting evidence that the cell-matrix adhesion properties of ILK are key to its active participation in therapeutic resistance. In breast cancer, the presence of soft tissues, relative to the primary tumor, at sites of distant metastasis is associated with increased autophagy and downregulation of estrogen receptor alpha (ERα) expression, resulting in resistance to tamoxifen [29] and enabling dormancy.

There is a particularly close relationship between ILK-mediated signaling and sensitivity to platinum-based chemotherapeutics such as cisplatin, especially in ovarian and lung cancer. The mechanisms of ILK-mediated regulation are associated with alteration in cell adhesion and EMT-mediated plasticity. For example, studies evaluating the efficacy of tripterygium glycosides (GTW) as an adjuvant therapeutic agent (anti-cancer agent) in chemo-resistant A2780/DPP epithelial ovarian cancer (EOC) cells, together with cisplatin (DPP), showed an association between activation of ILK signaling, EMT and chemoresistance [58]. While GTW inhibited proliferation, migration, and invasion of these cells, and intensified cisplatin sensitivity, the combination of GTW and cisplatin inhibited expression of N-cadherin, ILK, phosphorylated (p)-Akt, p-GSK3β, and Slug, and increased E-cadherin levels, suggesting inhibition of EMT via the ILK/Akt/GSK3β/Slug pathway [58]. In vivo, the combination reduced tumor burden, increased survival, and regulated EMT through the ILK/Akt/GSK3β/Slug pathway [58].

ILK factors prominently in the progression of lung adenocarcinoma [71]. ILK, together with PINCH1 and β-parvin (IPP complex), is overexpressed in human lung adenocarcinoma samples and in KRAS-driven disease in mice [71]. Overexpression of ILK in lung cancer cells results in the upregulation of PINCH1, β-parvin, and Ras suppressor protein 1 (RSU1) expression, while pharmacologic inhibition of ILK in KRAS-mutant lung cancer cells suppressed cell growth, migration, and EMT [71]. Pharmacologic inhibition of ILK in KRAS-mutant lung cancer cells increases sensitivity to platinum-based chemotherapy [71]. Furthermore, overexpression of ILK and Src homology 2 (SH2) domain–containing phosphatase 2 (SHP2) is associated with poor outcomes in patients with epidermal growth factor receptor (EGFR)-positive non-small cell lung cancer (NSCLC) who receive monotherapy with EFGR tyrosine kinase inhibitors (TKIs), suggesting both a potential avenue for stratification of patients with EGFR mutations and a rationale for combinatorial approaches using EGFR TKIs and targeted inhibitors of ILK and SHP2 [72].

In addition to resistance to pharmacologic agents, studies point to a contribution of ILK in resistance to radiotherapy. For example, in the context of gliobastoma multiforme (GBM), radioresistant p53-wildtype, but not p53-mutant GBM cells are sensitized to radiotherapy in the context of genetic depletion of PINCH or ILK, identifying an ILK-dependent therapeutic vulnerability to radiation treatment in these cells [73]. Whether other tumor types exhibiting p53-wildtype status are similarly sensitive to targeting ILK in combination with radiotherapy awaits further investigation.

Recent work also suggests that ILK is involved in the development of immune tolerance in CMS4, a mesenchymal subtype of colorectal cancer associated with poor prognosis and chemoresistance. ILK was identified as a proximal effector of PrPC, a cellular prion protein overexpressed in CMS4 tumors and tasked with phenotypic maintenance [74]. The PrPC-ILK signaling axis regulates the expression and activity of the tryptophan-metabolizing enzyme Indoleamine-2,3-dioxygenase 1 (IDO1), thereby regulating immune tolerance [74].

## 6. The Role of ILK in Leukemia and Resistance to TKIs

There is an emerging role of ILK-mediated signaling in acute and chronic myeloid leukemia (CML). It is known that leukemic stem cells (LSCs) reside in a protective niche in bone marrow, that these cells interact with their niche environment through FA, and that these are the key cells responsible for failure of therapy and patient relapse. ILK is upregulated in CML progenitors and LSCs, particularly in patients resistant to TKIs such as imatinib and dasatinib [75]. Depletion of ILK expression or pharmacologic inhibition of ILK activity in LSCs decreases growth, sensitizes TKI-resistant cells to targeted therapy and impairs LSC self-renewal in vivo [75]. Importantly, targeting LSC ILK activity with the small molecule QLT-0267 inhibits p-GSK3β (S9) and p-Akt (S473), as well as p-signal transducer and activator of transcription 3 (p-STAT3) (Y705), sensitizes these cells to TKIs, and when used in combination, shows strong synergistic effects with no adverse effects on normal BM cells, demonstrating cancer-selective targetability [75] Transcriptomic and functional analyses revealed that pharmacologic inhibition of ILK kinase activity targets quiescent LSCs from TKI non-responders by downregulating oxidative phosphorylation and ROS [75], identifying a metabolic vulnerability of ILK inhibition. Furthermore, combination treatment using QLT-0267 and dasatinib increases survival in xenograft models of advanced CML and reduces long-term engraftment of human LSCs in patient-derived xenograft (PDX) models using primary CML patient samples, [75], implicating ILK as an adjuvant target in CML [75].

Studies using models of imatinib-resistant CML have also demonstrated the presence of altered interactions with the bone marrow microenvironment (BMM) involving a fibronectin/β3-integrin/ILK pathway. While β3-integin and ILK expression were increased in imatinib-resistant disease, the integrin-ILK interaction is impaired and results in ILK-dependent decreases in fibronectin deposition [76]. Co-treatment of imatinib-resistant disease with an ILK inhibitor and ponatinib results in increased survival, concomitant with increased fibronectin deposition and β3-integrin expression, suggesting that modulating these interactions in the BMM influence leukemia progression and clinical outcome in TKI-resistant CML in vivo [76]. Collectively, these studies demonstrate the importance of ILK in controlling LSCs in the bone marrow niche to promote CML and demonstrate that specific targeting of ILK may be beneficial in providing clinical impact in the setting of TKI-resistant disease.

Similarly, ILK controls several signaling pathways that are often aberrantly regulated in acute myeloid leukemia (AML), arguing for a potential role for ILK in AML progression [77]. ILK is constitutively expressed by AML blast cells and ILK-mediated phosphorylation of Akt and GSK3β downstream of PI3K pathway activation of fms-like tyrosine kinase 3 (FLT-3), a receptor tyrosine kinase (RTK) critical for AML LSC maintenance, controls AML cell survival and proliferation [77]. Pharmacologic inhibition of ILK reduces Akt phosphorylation induced by stromal cells in the BMM and suppresses leukemia in the bone marrow, suggesting that ILK activity is critical to disease progression. ILK signaling can also mediate increases in inflammatory cytokine production and may drive immune evasion in AML [77]. ILK signaling is involved in mediating an interleukin-6 (IL-6)/STAT3/NF-κB feed-back loop that drives ILK expression and IL-6 production by AML cells, and ILK activity and expression are associated with the production of IL-1, which is enriched in AML and induces the growth of LSCs. While the intricacies of ILK signaling in AML require further investigation, its role both in cell survival and immune invasion highlights its potential as a cancer-specific target in this context.

## 7. Outstanding Questions

In this perspective, we have discussed recent developments that place ILK and its interactors as critical regulators of various steps of the metastatic cascade. Much of what we have discussed implicates ILK complexes as critical nodes in various signaling pathways. While the IPP complex undoubtedly plays a role as a mechanotransduction/adaptor complex to facilitate signaling, as described above, there are major gaps in our understanding of the precise molecular basis of the role of IPP in signaling. For example, while it is known that knockdown of PINCH and Parvin impacts downstream signaling of Akt, depletion of these proteins also impacts ILK stability, and the direct role of PINCH and Parvin, independent of ILK, has not been explored. In addition, Parvin has been shown to regulate ILK activity [78], again highlighting the necessity of investigating the contribution of IPP proteins independently of ILK. This gap is hampering the development of potential therapeutic strategies to block IPP-mediated signaling and inhibit metastasis.

On the other hand, ILK has been shown, in hundreds of papers, to promote the phosphorylation of proteins and kinases that regulate key signaling nodes. ILK consists of an atypical kinase domain, and as such has been considered to be a pseudokinase [79]. However, highly purified recombinant ILK has been unequivocally demonstrated to have protein kinase activity [19,78,80,81,82] and small molecule inhibitors that bind to the kinase domain have been identified [9,83,84,85,86,87]. The relegation of ILK as a pseudokinase has resulted in a generalized lack of interest in the development of agents targeting ILK directly. Many other “atypical ” kinases, such as DNA-dependent protein kinase, catalytic subunit (DNA-PKcs), ataxia telangiectasia mutated (ATM), ataxia telangiectasia and Rad3-related (ATR), and mTOR, belong to a subgroup of the “atypical” eukaryotic protein kinase family [88], but continue to be considered as bona fide kinase targets with the development of potent inhibitors that have progressed to the clinic [89].

Here we have discussed the extensive recent literature that implicates ILK in tumor progression, metastasis, and drug resistance. The continued demonstration of ILK as a validated therapeutic target and the demonstration of its kinase activity warrants a greater effort in identifying and developing potent “druggable” compounds to inhibit ILK activity. In addition to such compounds having direct effects on ILK activity and downstream signaling, they could also be useful as critical agents for the development of targeted degradation of ILK through the proteolysis targeting chimera (PROTAC) technology platform [90], offering alternative strategies to target this important protein.

## 8. Conclusions

Since its initial identification in 1996 [91], ILK continues to be of interest with the publication of over 1800 papers describing various roles in development, tissue homeostasis, and disease. In particular, ILK’s role in tumor progression has been solidified and ILK is considered to be a validated target for cancer therapy.

In this perspective, we have highlighted recent developments that strongly implicate ILK in the various phases of the metastatic cascade, and in resistance to chemo- and targeted therapeutic agents. We hope that many of these compelling and intriguing findings will trigger further interest in identifying strategies to inhibit ILK function to overcome metastasis and drug resistance.

## Data Availability

The data presented in this study are available on request from the corresponding author.
